# Path analysis of biopsychosocial correlates of physical activity among patients with colorectal cancer

**DOI:** 10.1038/s41598-025-25533-z

**Published:** 2025-11-24

**Authors:** Qian Rao, Lixia Zhang, Yongdong Jin, Hao Zhang, Jun lv, Dan Li, Yuping Zhang

**Affiliations:** 1 Department of Hepatobiliary and Pancreatic Surgery, Sichuan Clinical Research Center for Cancer, Sichuan Cancer Hospital & Institute, Sichuan Cancer Center, Affiliated Cancer Hospital of University of Electronic Science and Technology of China, Chengdu, China; 2 Department of Abdominal Oncology, Sichuan Clinical Research Center for Cancer, Sichuan Cancer Hospital & Institute, Sichuan Cancer Center, Affiliated Cancer Hospital of University of Electronic Science and Technology of China, No. 55, Section 4, Renmin South Road, Wuhou District, Chengdu, 610041 Sichuan China; 3 School of Nursing, Chengdu Medical College, Chengdu, China

**Keywords:** Colorectal cancer, Physical activity, Psycho‐oncology, Biopsychosocial, Path analysis, Colorectal cancer, Psychology

## Abstract

This study explored the underlying pathways linking physical activity (PA) and associated factors among Chinese patients with colorectal cancer (CRC) using path analysis. Although the benefits of PA in cancer care are well established, participation rates among CRC patients in China remain unclear, and influencing pathways are not fully understood. A cross-sectional survey was conducted with 211 inpatients at a tertiary cancer hospital in Southwest China from January to June 2024. Data on PA levels, fatigue, anxiety, depression, perceived exercise barriers and benefits, social support, and exercise self-efficacy were collected. The hypothesized model was tested using Mplus version 8.3. The median PA level was 3066 MET-min/week (i.e., metabolic equivalent minutes per week; IQR: 1584–4914), with 56.87% meeting the American College of Sports Medicine’s guidelines. The model showed good fit: χ2/df = 1.418, CFI = 0.988, TLI = 0.974, RMSEA = 0.045, and SRMR = 0.021. Fatigue, perceived barriers, and exercise self-efficacy showed direct associations with PA, whereas anxiety, depression, and perceived benefits related indirectly via self-efficacy and/or social support. Our path analysis disentangles direct vs indirect links, pinpoints self-efficacy as the key modifiable target, and highlights fatigue and perceived barriers as priority levers—informing multidimensional, targeted interventions.

## Introduction

Colorectal cancer (CRC), ranking second in mortality and third in incidence among cancers worldwide, is the most prevalent malignant tumor of the digestive tract^1^. Its onset is concealed and tends to be younger. By the time most patients are diagnosed, the disease has often progressed to middle or late stages^2^. The comprehensive treatment approach, primarily centered on surgery and supplemented by radiotherapy, chemotherapy, immunotherapy, and targeted therapy, plays a crucial role in extending patients’ survival. Nevertheless, various factors—including the disease itself, adverse treatment reactions, the impact of a stoma on daily life, and the stigma associated with the disease—significantly impair patients’ physiological (i.e., physical/clinical), psychological, and social functions. These impairments lead to restrictions in their activities, permanent alterations in their movement patterns, and ultimately insufficient physical activity (PA)^3,4^.

Numerous studies have demonstrated that the PA levels of CRC patients are closely related to health outcomes, underscoring the critical importance of improving their PA levels^5–8^. Physical activity is defined as any bodily movement generated by skeletal muscles that requires energy expenditure, according to the World Health Organization^9^. Previous research has shown that PA levels among CRC patients are associated with demographic and clinical factors such as age, occupation, marital status, smoking history, disease stage, comorbidities, and BMI^10–15^.

More importantly, grounded in the biopsychosocial model^16^, mounting evidence indicates that physiological, psychological, and social factors are associated with PA levels among CRC survivors^17,18^. The biopsychosocial model posits that health and illness result from the complex interaction of biological, psychological, and social factors. Unlike traditional biomedical models that focus primarily on biological causes, the biopsychosocial model recognizes the contribution of psychological states (e.g., mental health, stress) and social influences (e.g., social support, cultural norms) to health behaviors. Furthermore, the COM-B model^19^— a core component of the Behavior Change Wheel—conceptualizes behavior as resulting from three interacting components: capability, opportunity, and motivation. Capability encompasses the physical and psychological skills, knowledge, and attributes necessary for performing a behavior. Motivation refers to cognitive processes that energize and direct behavior; and opportunity denotes external factors that enable or prompt the behavior. According to COM-B, enhancing an individual’s physical and psychological capability, providing adequate opportunity/support, and fostering motivation can facilitate adoption and maintenance of healthy behaviors. While the relationship between PA and biopsychosocial domains is well established, further work is needed to clarify the pathways linking these domains to PA and to translate these insights into targeted interventions. Understanding these pathways will allow us to identify specific, modifiable drivers of PA and enhance the effectiveness of interventions by tailoring them to address the precise mechanisms that hinder or promote PA.

Fatigue, anxiety, and depression have been shown to significantly affect PA levels^13,20,21^. Fatigue, a prevalent and non-negligible symptom among CRC survivors, has been identified as a significant predictor of PA levels^22^. Additionally, fatigue often co-occurs with psychological risk factors such as anxiety and depression^23^. However, the precise mechanisms by which these factors affect PA remain unclear.

Perceived exercise benefits and barriers also play important roles in shaping PA levels^13,17^. Based on the Health Promotion Model^24^, individuals with higher perceived benefits and lower perceived barriers are more likely to adopt and sustain PA. Nevertheless, the specific pathways through which perceived exercise benefits and barriers influence PA require further investigation. The effect of these perceptions is further influenced by self-efficacy for exercise, which determines whether individuals feel capable of overcoming these barriers and engaging in PA.

Self-efficacy for exercise, defined as an individual’s belief in their ability to successfully perform exercise-related activities^25^, is a key determinant of exercise adherence and behavior^4^. Bandura’s self-efficacy theory suggests that self-efficacy is influenced by factors such as physical condition, emotions, past experiences, and social support^26^. It also mediates the relationship between social support and PA, as demonstrated by a meta-analysis showing that social support indirectly influences exercise behavior through self-efficacy^27^. As self-efficacy influences how individuals respond to challenges, social support plays an important role in either reinforcing or undermining one’s confidence and, consequently, their ability to engage in PA.

Social support, as an external resource, plays a crucial role in maintaining physical and mental well-being^28^. Meta-analyses have highlighted its significant role in promoting PA^29^. According to social support theory, the level of social support is influenced by individual, developmental (past experiences and perceptions), and environmental factors^30^. This suggests that fatigue, anxiety, depression, perceived exercise benefits, and perceived exercise barriers can impact an individual’s level of social support.

The biopsychosocial model provides a foundational framework for understanding the multifaceted nature of health behaviors, emphasizing the interplay of physiological, psychological, and social factors in shaping PA among CRC patients. Building on this foundation, the Behavior BCW model offers a structured approach for identifying the key components—capability, opportunity, and motivation—that influence behavior. Meanwhile, social support theory and self-efficacy theory help to elucidate the psychosocial mechanisms through which these factors interact to impact PA engagement.

Extensive research has been conducted on the factors influencing PA. However, research specifically focusing on the current levels of PA and the associated factors among CRC patients remains limited. Moreover, most existing research has employed traditional statistical approaches that examine only one or two variables at a time, thereby failing to capture the complex interrelationships among multiple biopsychosocial factors. This limitation may constrain the development of effective PA interventions. Understanding the specific pathways through which biopsychosocial factors influence PA behavior is critical for identifying modifiable determinants and selecting the most appropriate intervention targets. By elucidating these mechanisms, interventions can be more precisely tailored to address the psychological, social, or physical barriers most relevant to specific patient populations, thus enhancing their potential to effect sustained behavior change.

Therefore, this study integrates common biopsychosocial variables related to PA and provides a detailed explanation of the relationships and path coefficients in the model. The goal is to enhance our understanding of these mechanisms and offer insights for designing future exercise interventions. Specifically, this study aims to (1) assess the current levels of PA among CRC patients and (2) clarify the hypothesized pathways through which various factors are associated with PA. Based on these theoretical foundations and previous literature, we hypothesize that fatigue, anxiety, depression, and perceived exercise benefits/barriers influence PA levels through social support and self-efficacy for exercise.

## Methods

### Study design and setting

This cross-sectional study was conducted between January and June 2024 using convenience sampling to consecutively recruit CRC patients receiving treatment at Sichuan Cancer Hospital & Institute in China. This hospital, the largest comprehensive center for tumor treatment, prevention, research, and education in Southwest China, serves patients nationwide with 1590 open beds, over 450,000 outpatient visits, and more than 59,000 inpatient admissions annually.

### Participants and sample size

Determining an appropriate sample size is critical in path analysis to ensure the reliability and stability of parameter estimates. According to established guidelines, a widely accepted heuristic suggests that at least 10 observations per estimated parameter are necessary^31^. Given that the model under consideration included 18 paths, the recommended minimum sample size was 180. Considering the model’s complexity, including 8 observed variables, a sample size between 80 and 160 observations was deemed advisable to adequately capture data variability. Structural Equation Modeling (SEM) literature frequently recommends a minimum sample size of 200 for robust and reliable estimates, especially in more complex models^32^. Therefore, a minimum sample size of 200 was determined for this study.

### Recruitment procedure and data collection

The inclusion criteria were as follows: (1) pathological diagnosis of colon or rectal cancer; (2) age ≥ 18 years; and (3) provision of informed consent and voluntary participation. The exclusion criteria were: (1) less than 3 months post-tumor resection surgery; (2) physical disabilities or neuromuscular or joint diseases affecting mobility; (3) history of mental illness or communication barriers; and (4) severe organ dysfunction. Six well-trained postgraduate researchers distributed and collected the questionnaires. Prior to participation, all participants were provided with detailed information regarding the purpose and content of the study, and written informed consent was obtained from each participant. To minimize the impact of treatment factors on patient activity, patients who were less than three months post-surgery were excluded, and chemotherapy patients were invited to respond during their chemotherapy intervals. To ensure the quality of questionnaire completion, research team members accompanied patients individually to address questions and concerns throughout the process. For patients with literacy challenges, researchers read the questions verbatim and recorded their responses. In total, 220 patients were surveyed. Of these, three withdrew from the study, two provided invalid responses, and data were missing for four patients. Consequently, 211 valid questionnaires were included in the final analysis, yielding an effective response rate of 95.9%. This study was approved by the Medical Research Ethics Committee of Sichuan Cancer Hospital (Approval No. SCCHEC-02–2024-014) and was conducted in accordance with the principles of the Declaration of Helsinki.

### Measurement

Demographic and clinical characteristics were collected through patient self-report and verified via chart review. Demographic variables included age, sex, marital status, educational level, employment status, economic status, and type of health insurance used to pay for treatment (i.e., payment method). Clinical characteristics included surgical history, receipt of chemotherapy or radiotherapy, disease stage, presence of a stoma, Charlson Comorbidity Index^33^, and BMI.

The International Physical Activity Questionnaire-Short Form (IPAQ-SF**)** was used to assess respondents’ PA levels over the past seven days, and its validity has been well established^34^. The questionnaire consists of 7 items that evaluate different intensities of PA—vigorous, moderate, walking, and sedentary—each assigned a Metabolic Equivalent of Task (MET) value of 8.0, 4.0, 3.3, and 1.1, respectively. An individual’s PA level is calculated by multiplying the MET value by the reported duration of the activity (minutes per day). The overall PA level is determined by summing the values for vigorous, moderate, and walking activities.

The Brief Fatigue Inventory (BFI), developed by the Pain Research Team at the Anderson Cancer Center in 1999^35^, is widely used to assess fatigue in cancer patients due to its straightforward design and high reliability. The scale comprises 9 items to evaluate the severity of fatigue symptoms and their impact on daily life. Scores range from 0 to 10, with higher scores indicating greater fatigue severity. The Chinese version of the BFI has demonstrated good reliability and validity^36^.

The Hospital Anxiety and Depression Scale (HADS), extensively used in hospital settings, assesses anxiety and depression levels among patients^37^. The scale consists of two subscales: Anxiety (HADS-A) and Depression (HADS-D), each comprising 7 items, for a total of 14 items rated on a 4-point Likert scale. Higher scores indicate more severe anxiety or depression.

The Exercise Benefits/Barriers Scale (EBBS) evaluates perceived benefits and barriers to exercise. The scale consists of 43 items divided into two subscales: Perceived Exercise Benefits (29 items) and Perceived Exercise Barriers (14 items)^24^. The benefits subscale assesses five dimensions: life enhancement, physical performance, psychological outlook, health prevention, and social interaction. The barriers subscale evaluates four dimensions: physical exertion, exercise environment, time expenditure, and family discouragement. The scale employs a 4-point Likert scale, where higher scores indicate greater perceived benefits and fewer barriers to exercise. The Chinese version of the EBBS has shown excellent reliability and validity^38^.

Social support was measured using the Perceived Social Support Scale (PSSS)^39^. The PSSS comprises 12 items across three dimensions: family support, friend support, and other support. It uses a 7-point Likert scale, with higher scores reflecting greater perceived social support. The scale demonstrates strong reliability and validity and has been widely used in studies involving Chinese populations^40^.

The Self-Efficacy for Exercise Scale (SEE) was used to assess individuals’ confidence in completing exercise tasks during PA^41^. This scale includes 9 items rated from 0 (“no confidence at all”) to 10 (“extremely confident”), yielding a total score range of 0 to 90. Higher scores indicate greater self-efficacy for exercise. The Chinese version of the SEE has demonstrated excellent reliability and validity^42^.

### Statistical analysis

SPSS for Windows, version 23.0, was used to calculate descriptive statistics for summarizing the demographic characteristics of the study participants and to perform correlation analyses between research variables. To ensure effective alignment between PA level groupings and individual health benefits, this study followed the data truncation guidelines established by the International Physical Activity Questionnaire (IPAQ) Working Group, addressing any exceptionally high values accordingly^34^. Categorical variables were presented as frequencies and percentages. Continuous variables were described as means and standard deviations (SD) for normally distributed data, and as medians and interquartile ranges (IQR) for non-normally distributed data. Spearman’s rank correlation coefficient was used to assess the relationships between variables.

Path model testing was conducted using Mplus, version 8.3. Prior to testing model fit, a common method bias test was performed in SPSS to check for systematic errors potentially caused by the data collection method^43^. The overall model fit was evaluated using multiple fit indices, including the chi-square test (χ2/df < 3.0), comparative fit index (CFI > 0.90), Tucker-Lewis index (TLI > 0.95), root mean square error of approximation (RMSEA < 0.08), and standardized root mean square residual (SRMR < 0.05)^44,45^. The maximum likelihood method was used to estimate model parameters. Given the deviation from normality in the PA data, a logarithmic transformation was applied to approximate a normal distribution. Standardized beta coefficients were reported, as they help identify the relative influence of variables on the endogenous variables. Through iterative refinement, non-significant paths were removed to derive an optimized path model. In this study, the bootstrap confidence interval (CI) was set at 95%, with 5,000 bootstrap samples. A mediating effect was considered significant if the 95% CI did not include zero. Statistical significance was defined as a p-value < 0.05.

## Results

### Participant characteristics

The descriptive characteristics of the participants are summarized in Table 1. The mean age of the patients was 57.51 ± 9.03 years. More than half of the participants were male (58.77%) and had an education level of junior high school or below (54.03%). The majority were married (94.79%) and unemployed (81.99%). Additionally, 99.05% had some form of medical insurance coverage (including public health insurance, commercial insurance, or a combination of both); however, 85.3% reported experiencing financial burdens. Furthermore, 71.56% had undergone surgery, and 96.21% had received chemotherapy. Regarding cancer severity, 90.52% of the patients were classified as stage III or IV. The mean BMI was 23.00 ± 3.28, and the median Charlson Comorbidity Index was 1 (IQR: 1–2).

**Table 1:** Sample characteristics (n = 211).

Variables	n	%
Sex		
Male	124	58.77
Female	87	41.23
Marital status		
Married	200	94.79
Unmarried/divorced/widowed	11	5.21
Education levels		
Junior school or less	114	54.03
High school/technical secondary	45	21.33
College or above	52	24.64
Occupation		
Manual labor	9	4.27
Mental labor	19	9.00
Manual and mental labor	10	4.74
None	173	81.99
Financial strain		
None	31	14.69
Mild or moderate	89	42.18
Serious	91	43.13
Operation treatment		
Yes	151	71.56
No	60	28.44
Chemotherapy		
Yes	203	96.21
No	8	3.79
Have stoma		
Yes	39	18.48
No	172	81.52
BMI		
< 18.5	15	7.11
18.5–24.0	123	58.29
> 24.0	73	34.60

### Physical activity among CRC patients

The PA levels among CRC patients are summarized in Table 2. The median total energy expenditure from PA was 3066 MET-min/week, with a range from 0 to 15,225 MET-min/week. The IQR was 1584 to 4914 MET-min/week. Among the participants, 8.06% engaged in high-intensity PA, 45.50% in moderate-intensity PA, and 46.45% participated only in walking. Additionally, 120 patients (56.87%) met the American College of Sports Medicine (ACSM) recommendations of at least 150 min of moderate-intensity or 75 min of high-intensity PA per week. Notably, 66 patients (31.28%) did not engage in any moderate- to high-intensity PA. Furthermore, the median weekly sedentary time was 1680 min.

**Table 2:** Physical activity scores of different intensities in patients with colorectal cancer (n = 211).

Variables		Minimum	Maximum	Median (Q1-Q3)
Vigorous-intensity PA	Time (min/week)	0	1260	0 (0–0)
	(MET-min/week)	0	10,080	0 (0–0)
Moderate-intensity PA	Time (min/week)	0	1260	210 (0–630)
	(MET-min/week)	0	5040	840 (0–2520)
Walking	Time (min/week)	0	1260	450 (280–840)
	(MET-min/week)	0	4158	1485 (924–2772)
Total PA score (MET-min/week)		0	15,225	3066 (1584–4914)
MVPA time (min/week)		0	1680	210 (0–630)
Sedentary time (min/week)		105	4200	1680 (840–2100)

*MVPA* Moderate to vigorous physical activity.

### Common method variance assessment

This study employed self-report measures to collect data, which may introduce the issue of common method variance (CMV). To address this concern and enhance the rigor of the study, Harman’s single-factor test was performed prior to data analysis to assess the presence of CMV. The results revealed 16 factors with eigenvalues greater than 1, and the variance explained by the first factor was 36.02%, which is below the critical threshold of 40%. Therefore, no significant common method variance was detected in this study^43^.

### Correlations among study variables

Spearman’s correlation analysis was conducted to examine the relationships between key variables and PA. The results revealed significant correlations between PA and fatigue (r = −0.281, p < 0.01), anxiety (r = −0.144, p < 0.05), depression (r = −0.185, p < 0.01), perceived exercise barriers (r = 0.262, p < 0.01), perceived exercise benefits (r = 0.225, p < 0.01), and self-efficacy for exercise (r = 0.389, p < 0.01). However, social support was not significantly correlated with PA (r = 0.088, p > 0.05) (see Table 3).

**Table 3:** Correlations matrix for variables (n = 211).

Variables	1	2	3	4	5	6	7	8
Fatigue	1.00							
Anxiety	0.645**	1.00						
Depression	0.705**	0.733**	1.00					
Exercise barriers	−0.556**	−0.515**	−0.474**	1.00				
Exercise benefits	−0.348**	−0.403**	−0.303**	0.694**	1.00			
Social support	−0.356**	−0.294**	−0.366**	0.344**	0.438**	1.00		
Self−efficacy for exercise	−0.422**	−0.445**	−0.333**	0.768**	0.669**	0.425**	1.00	
Physical activity	−0.281**	−0.144*	−0.185**	0.262**	0.225**	0.088	0.389**	1.00

p < 0.05 was considered statistically significant; *p < 0.05, **p < 0.01.

### Path analysis

Through stepwise modifications based on the Modification Index (MI), non-significant paths were removed, resulting in the final path analysis model for factors influencing PA^46^. The overall fit indices indicated a good model fit: χ2/df = 1.418 (< 3.0), CFI = 0.988 (> 0.90), TLI = 0.974 (> 0.95), RMSEA = 0.045 (< 0.08), and SRMR = 0.021 (< 0.05).

**Fig. 1:**
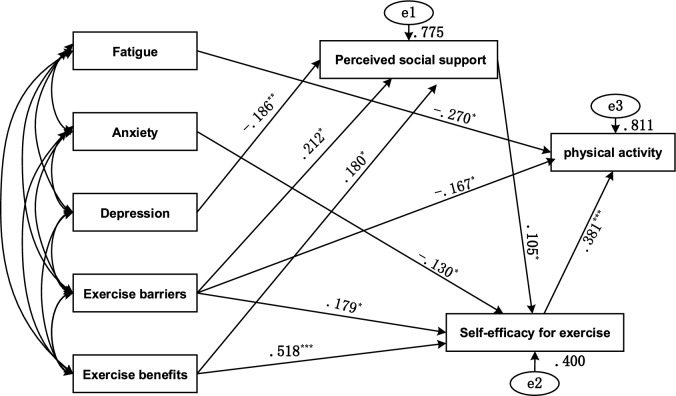
Path analysis of biopsychosocial correlates of physical activity. * p < 0.05, ** p < 0.01, and *** p < 0.001.

Figure 1 presents the standardized path coefficients for the final model. The path analysis results reveal that fatigue, perceived exercise barriers, and self-efficacy for exercise have direct effects on PA. Anxiety, depression, perceived exercise barriers, and perceived exercise benefits indirectly influence PA through perceived social support, self-efficacy for exercise, or both.

Table 4 displays the standardized direct, indirect, and total effects on PA among CRC patients. Notably, the total effect of perceived exercise barriers on PA was not statistically significant due to a suppression effect. Specifically, the direct effect of perceived exercise barriers on PA was significant and negative (β = −0.167, 95% CI [−0.313 to −0.001], p = 0.035), while the indirect effect, mediated through perceived social support and/or self-efficacy for exercise, was positive and significant (β = 0.067, 95% CI [0.022 to 0.157], p = 0.029). This opposing influence resulted in a non-significant total effect (β = −0.090, 95% CI [−0.232 to 0.089], p = 0.267), with a suppression effect of 0.01. Ranked by the absolute value of the total effect in descending order, the key variables in the paths are as follows: self-efficacy for exercise (0.381) > fatigue (−0.270) > perceived exercise benefits (0.205) > perceived exercise barriers (−0.090) > anxiety (−0.050) > perceived social support (0.040) > depression (−0.007). Except for perceived exercise barriers, all other paths had 95% confidence intervals excluding 0, indicating statistical significance.

**Table 4:** Standardized direct, indirect, and total effects of all study pathways (n = 211).

Paths	Direct effects	Indirect effects	Total effects
Fatigue → PA	−0.270 (−0.478, −0.035)	−	−0.270 (−0.478, −0.035)
Anxiety → PA	−	−0.050 (−0.122, −0.010)	−0.050 (−0.122, −0.010)
Depression → PA	−	−0.007 (−0.022, −0.001)	−0.007 (−0.022, −0.001)
PSS → PA	−	0.040 (0.008, 0.086)	0.040 (0.008, 0.086)
SEE → PA	0.381 (0.179, 0.558)	−	0.381 (0.179, 0.558)
Exercise barriers → PA	−0.167 (−0.313, −0.001)	0.076 (0.022, 0.157)	−0.090 (−0.232, 0.089)
Exercise benefits → PA	−	0.205 (0.098, 0.313)	0.205 (0.098, 0.313)

*PA* physical activity, *PSS* perceived social support, *SEE* self-efficacy for exercise.

## Discussion

This study assessed the PA status of Chinese CRC patients and explored the biopsychosocial pathways underlying PA through path analysis. The results revealed that fatigue, perceived exercise barriers, and self-efficacy for exercise had direct effects on PA levels. Meanwhile, anxiety, depression, perceived exercise benefits, and perceived exercise barriers were indirectly associated with PA through the mediating effects of perceived social support and/or self-efficacy. These findings underscore the complex interplay of psychological, social, and physical factors in shaping PA behaviors among CRC patients.

In our sample, 56.87% of participants met the ACSM guideline for MVPA, with a median total energy expenditure of 3066 MET-min/week. According to ACSM standards, moderate-intensity PA generally corresponds to 3.0–5.9 METs and vigorous-intensity to ≥ 6.0 METs, with ≥ 600 MET-min/week considered the minimum recommended threshold. Thus, participants demonstrated PA levels well above the benchmark. These findings help enrich our understanding of PA levels among Chinese CRC patients in the context of current evidence. Although higher than some China-based studies^4,13^, our estimates align with international cohorts^11,47,48^. This discrepancy may result from differences in measurement tools, national health policy efforts, or sample characteristics. We used the IPAQ-SF, which has cross-cultural validity and allows comprehensive assessment of PA domains, facilitating standardized data collection.

Moreover, recent public health campaigns in China—particularly the “Healthy China 2030” initiative—may have promoted greater awareness and engagement in PA through medical systems and media^49^. Notably, 99.05% of participants had medical insurance, but 85.3% still reported financial burdens. Most were unemployed (81.99%) and middle-aged or older, and many likely maintained PA through routine tasks such as housework or caregiving rather than structured exercise^50^. Indeed, total PA energy expenditure ranged widely (0 to 15,225 MET-min/week), and only 8.06% engaged in vigorous activity, whereas 31.28% reported no MVPA. These variations underscore substantial disparities in PA and sedentary behavior in this population.

Given the inverse relationship between PA and mortality and the positive association between sedentary behavior and all-cause mortality in CRC patients^5,51^, these findings highlight a need for targeted clinical interventions. Education on the benefits of PA and risks of sedentary behavior—combined with feasible, individualized activity plans—may support CRC patients in maintaining adequate PA levels, particularly those with low activity levels or prolonged sedentary time.

Path analysis showed that self-efficacy for exercise was positively and significantly associated with PA and was the strongest correlate. This finding aligns with meta-analytic results^17^ identifying self-efficacy for exercise as a key factor in enhancing individuals’ adherence to exercise. Individuals facing challenges are more likely to persevere if they have high self-efficacy for exercise because they believe in their ability to overcome obstacles. Furthermore, the results reveal the mediating role of self-efficacy for exercise, demonstrating its mediation between anxiety, depression, perceived exercise barriers, perceived exercise benefits, perceived social support, and PA. This further suggests a close link between biopsychosocial factors and PA through self-efficacy for exercise. Beyond Bandura’s theory of self-efficacy, several other theories also explore the relationships between biopsychosocial factors, self-efficacy, and PA, including Self-Determination Theory and the Theory of Planned Behavior^52,53^. Future research should integrate multiple theoretical frameworks to develop comprehensive intervention measures tailored to individuals, with a focus on testing effective strategies to enhance self-efficacy, aiming for more successful interventions to improve PA.

Path analysis indicated that fatigue had the second-largest total association with PA; higher fatigue levels were associated with lower PA. Numerous studies have also confirmed the negative impact of fatigue on PA^18,22^, primarily due to the extensive influence of cancer-related fatigue on physiological, psychological, and social functions^54^. Therefore, alleviating fatigue symptoms is crucial for improving PA levels during the rehabilitation process of cancer patients. This study hypothesized that fatigue could indirectly affect PA levels through the mediating roles of social support and self-efficacy for exercise. However, contrary to expectations, the results indicate that fatigue does not affect PA through these mediators, but rather directly influences PA. One possible reason fatigue did not affect PA through social support could be the use of the PSSS in this study, which focuses more on assessing an individual’s psychological health rather than the practical aspects of social support. In some cases, even if individuals experience fatigue, their emotional state may not significantly impact their perception or willingness to accept support from others. They may still perceive and accept support, thus maintaining a normal social support network. Similarly, the reason fatigue did not influence PA through self-efficacy for exercise may be linked to a cognitive dissociation between fatigue and self-efficacy. This belief is often based on past successes, knowledge, intrinsic motivation, or clear goal-setting. Therefore, even if individuals feel fatigued, they may still retain confidence in their exercise abilities, remaining relatively unaffected by temporary physical or psychological fatigue^55^..

Previous research has shown that perceived exercise benefits and perceived exercise barriers, as predictors of self-efficacy for exercise, are crucial factors in improving individual PA levels and in developing effective intervention strategies^56^. Perceived exercise barriers refer to an individual’s subjective perception and assessment of potential obstacles encountered during exercise. These perceived barriers can directly or indirectly influence the individual’s evaluation of the feasibility of exercise and, consequently, affect their behavior choices. Path analysis indicated that, due to suppressor effects, the total effect of perceived exercise barriers on PA was not statistically significant; however, both the direct and indirect effects were significant. Specifically, perceived exercise barriers affect PA through two indirect pathways: the first pathway suggests that perceived barriers weaken self-efficacy for exercise, which in turn leads to a decrease in PA levels; the second pathway shows that perceived barriers reduce an individual’s perceived social support, which subsequently undermines their confidence in their exercise abilities (i.e., self-efficacy), indirectly leading to lower PA levels. This finding underscores the need for a comprehensive, multidimensional approach to PA interventions. It is crucial not only to reduce individuals’ perception of exercise barriers but also to enhance their exercise cognition, increase self-efficacy, and expand social support networks, thus effectively promoting PA participation. Direct effect analysis revealed a negative correlation between perceived exercise barriers and PA, meaning that the fewer perceived barriers individuals report, the lower their PA levels tend to be. Possible explanations for this phenomenon include: (1) Reducing perceived exercise barriers may not directly increase PA levels but rather influences PA through the mediating effect of self-efficacy for exercise. If individuals lack confidence in their exercise ability (self-efficacy), even if perceived barriers decrease, their PA levels may not significantly increase; (2) PA levels are closely related to individual exercise habits and cognition. If individuals perceive their current level of PA as adequate or sufficient, they may not actively increase PA even if perceived barriers decrease. In conclusion, the influence of perceived exercise barriers on PA is complex and multidimensional. It involves not only direct effects but also indirect effects through mediators such as self-efficacy for exercise and perceived social support. Therefore, these factors should be considered comprehensively when developing intervention strategies.

Perceived exercise benefits showed the third-largest total association with PA, operating indirectly via self-efficacy and/or social support. Path analysis reveals that perceived exercise benefits do not directly affect PA but operate through two indirect pathways. The first pathway involves perceived exercise benefits enhancing self-efficacy for exercise, thereby indirectly promoting PA levels. This finding aligns with previous research^57^, which highlights the critical role of the positive impact of perceived exercise benefits on self-efficacy for exercise in improving individual PA. The second pathway suggests that perceived exercise benefits indirectly promote PA by enhancing both perceived social support and self-efficacy for exercise. This indicates that while perceived exercise benefits can shape positive attitudes toward exercise, these attitudes need to be mediated by factors such as increased self-efficacy for exercise and social support in order to translate into actual PA behavior. These findings suggest that interventions targeting PA should not only focus on enhancing individuals’ recognition of the benefits of exercise but also on strengthening their sense of self-efficacy and increasing social support from family members and healthcare professionals. This approach can effectively boost individuals’ motivation to engage in PA and promote higher levels of participation. Research also indicates that perceived exercise benefits are influenced by individuals’ ethnic and cultural backgrounds^58^. Therefore, when encouraging PA engagement, it is particularly important to adopt personalized, comprehensive, and multi-level intervention strategies.

Path analysis indicated that anxiety was indirectly associated with PA via exercise self-efficacy, with no significant pathway via social support. Previous research has primarily focused on the impact of PA on anxiety^59^; however, this study reveals, from a reverse perspective, how anxiety affects PA. These findings suggest that future research should place more emphasis on the impact of anxiety on PA. Regarding the result that anxiety did not influence PA through social support, we hypothesize that for CRC patients, anxiety is more often associated with concerns about their health and disease-related stress, which may not directly affect their perception of social support. Future studies could further explore how different levels of anxiety may influence social support, thereby providing a more comprehensive understanding of the relationship between the two.

The results suggested that depression was related to PA through a sequential, indirect pathway: higher depression was associated with lower perceived social support, which was associated with reduced exercise self-efficacy and, in turn, lower PA. A possible explanation is that depressive symptoms lead to negative emotional, cognitive, and behavioral effects, making it difficult for individuals to perceive or accept support from their social networks. This reduction in perceived support lowers individuals’ confidence in their ability to exercise. The decline in self-efficacy for exercise weakens their confidence, willingness, and enthusiasm to engage in physical activities, leading to a decrease in PA levels. These findings further elucidate the negative impact mechanisms of depression on PA, revealing the complex interactions between psychological factors, social support, and behavior. This discovery emphasizes the mediating roles of social support and self-efficacy for exercise between depression and PA, providing a theoretical basis for future intervention strategies.

This study has several limitations. First, the use of convenience sampling may have introduced selection bias and limited the generalizability of our findings. Participants were recruited from selected hospitals and may not fully represent the broader CRC population in China. Additionally, the demographic diversity of our sample could be improved—particularly in terms of occupational backgrounds and representation of patients with stomas—potentially affecting the variability of PA behaviors. Second, the cross-sectional design restricts the ability to draw causal inferences; future longitudinal studies are needed to assess temporal dynamics and causal pathways. Third, although we examined a broad range of influencing factors, the reliance on self-reported questionnaires may not fully capture participants’ actual behaviors or psychological states, introducing potential reporting bias. Fourth, we excluded patients who were within three months post-surgery or undergoing active chemotherapy in order to ensure data stability and construct validity. While this improved internal consistency, it may limit the generalizability of our findings to CRC patients undergoing acute treatment.

## Conclusion

The level of PA among CRC patients in China appears to have improved compared to previous studies, although substantial individual variability remains. The path analysis identified how physiological, psychological, and social factors influence PA, revealing both direct and indirect pathways. Specifically, fatigue, perceived exercise benefits, and exercise self-efficacy were found to directly affect PA levels, while anxiety, depression, perceived exercise benefits, and perceived exercise barriers exerted indirect effects through perceived social support, exercise self-efficacy, or both. These findings underscore the significance of integrating physiological, psychological, and social dimensions in understanding PA behaviors among CRC patients and highlight the need to account for these multidimensional factors when designing targeted intervention strategies.

## Data Availability

The data supporting the findings of this study are available from the corresponding author upon reasonable request.
